# CA 15-3, CRP, and LDH correlates with prognostic parameters in canine mammary neoplasms

**DOI:** 10.1590/1984-3143-AR2022-0086

**Published:** 2023-03-13

**Authors:** Breno Queiroz Pinheiro, Francisco Felipe de Magalhães, Francisco Wesley da Silva Alves, Isaac Neto Goes Silva, Augusto Manuel Rodrigues Faustino, Lúcia Daniel Machado da Silva

**Affiliations:** 1 Laboratório de Reprodução de Carnívoros, Faculdade de Veterinária, Universidade Estadual do Ceará, Fortaleza, CE, Brasil; 2 Laboratório de Patologia Clínica Veterinária, Faculdade de Veterinária, Universidade Estadual do Ceará, Fortaleza, CE, Brasil; 3 Departamento de Patologia e Imunologia Molecular, Instituto de Ciências Biomédicas de Abel Salazar, Universidade do Porto, Porto, Portugal

**Keywords:** cancer, dog, mammary gland, serum biomarker

## Abstract

The identification of putative prognostic factors in canine mammary neoplasms (CMNs) has been focused on tissue-specific biomarkers, but the serum biomarkers, including cancer antigen 15-3 (CA 15-3), c-reactive protein (CRP), and lactate dehydrogenase (LDH) have been demonstrated to display clinical application in cases of CMNs. The aim of the study was to evaluate the levels of these serum biomarkers and their association with well-established prognostic factors in CMNs. Samples from 15 female canines with CMNs and 15 clinically healthy ones were collected. The results were evaluated using the Tukey’s, Pearson, or Spearman tests. The cut-off point, sensitivity, specificity, and area under curve (AUC) were evaluated using the receiver operating characteristic (ROC) curve analysis in a logistic regression model (P<0.05). The levels of CA 15-3, CRP and LDH were significantly higher in the serum of female dogs with CMNs compared to the healthy ones. Moreover, these factors were positively correlated with ulceration, tumor size, histopathological grade, metastatic lymph node, and clinical staging. Female dogs with CMNs were found to exhibit highest serum levels of CA 15-3, CRP, and LDH. Therefore, they can be applied to improve the efficacy of the diagnosis and prognostic evaluation in casas of CMNs.

## Introduction

Female canine mammary neoplasms (CMNs) are defined as a heterogeneous group of diseases of complex classification, diagnosis, and prognosis. It is the most common type of tumors in female dogs and represents 50 to 70% of all types that affect this subset of the population (Cassali et al., 2020; Sorenmo et al., 2020).

In the past few decades, the identification of putative prognostic factors in CMNs has been focused on tissue biomarkers (Cassali et al., 2020). However, these biomarkers can only be evaluated after the surgical removal of the tumor, and thus are invasive, cumbersome, and undergo prolonged processing. On the other hand, serum biomarkers evaluation has advantages over tissue biomarkers, since it is a minimally invasive assessment procedure, and also exhibits dynamic changes according to the physiological and pathological states before the appearance of clinical symptoms (Kaszak et al., 2018).

In human breast cancer, the assessment of CA 15-3 (Darlix et al., 2016; Di Gioia et al., 2016; Shao et al., 2015), CRP (Allin and Nordestgaard, 2011; Han et al., 2011; Shimura et al., 2019), and LDH (Huang et al., 2016; Koukourakis et al., 2009; Naik and Decock, 2020) has been well described and correlated with prognostic factors and overall survival of patients. Recently, it was reported that the association of these biomarkers in women can improve the overall sensitivity of tumor detection, and its clinical application in follow-up examination was suggested (Di Gioia et al., 2016).

In veterinary medicine, CA 15-3 (Baba et al., 2019; Campos et al., 2012, 2015; Manuali et al., 2012), CRP (Planellas et al., 2009; Szczubiał et al., 2018), and LDH (Campos et al., 2012) have been suggested as good biomarkers of CMNs. Few biomarkers are specific for a single tumor type, but their association can increase diagnostic accuracy (Kabel, 2017). Some works suggest the combined dosage of CRP with other serum biomarkers increased the diagnostic accuracy for cancer detection (Ryu et al., 2019; Selting et al., 2016). In spite of these previous studies indicating an association of more than one biomarker in cases of cancer in dogs, there are no works evaluating the association of CA 15-3, CRP and LDH simultaneously in cases of CMNs.

Given the high incidence of CMNs and the need to standardize tests that are less cumbersome and allow serial analysis, we aimed in the current study to evaluate the combined serum levels of CA 15-3, CRP, and LDH in female dogs with CMNs, as well as their association with well-established prognostic factors.

## Methods

This study was approved by the Animal Experimentation Ethics Committee of the Ceará State University (UECE) (protocol no. 01047323/2019), and all dog owners who agreed to participate in the current study received proper orientation and signed a consent form.

### Animals

Thirty female dogs presented at the Veterinary Hospital of the Veterinary School of UECE were randomly selected and used without breed or age preference. After the clinical evaluation, the dogs were divided in two groups according to the following criteria: 15 with clinical and suggestive cytology diagnosis of CMNs with a mean age of 9.0 ± 2.0 (6 to 12 years in age); and 15 clinically healthy dogs showing no clinically apparent changes in the mammary gland or other systems with a mean age of 5.3 ± 3.0 (10 months to 11 years in age).

### Clinical examination

All dogs were subjected to an initial clinical examination consisting of visual inspection and palpation of all sets of mammary glands, regional lymph nodes. CBC, renal and hepatic serum evaluation were also performed. In the CMNs group, information about reproductive history, the number and measurement of tumors (by the largest tumor in cm), and the presence of ulceration were evaluated. Thoracic radiographs with three projections (ventrodorsal, right and left lateral-lateral), and total abdominal ultrasonography for metastasis screening were required to determine the TNM clinical staging following the guidelines of the World Health Organization (WHO) (Yamagami et al., 1996).

### Surgical removal

A variety of procedures were established based on the size, fixation to the surrounding tissue, and number of lesions when removing both tumors from the mammary gland and regional lymph nodes (inguinal and/or axillary) (Cassali et al., 2020).

### Histological examination

Samples were collected from neoplastic tissue, surrounding normal tissue and the regional lymph nodes. After the excision, the samples were fixed in 10% neutral formalin and embedded in paraffin after 24 h, sectioned at 4-μm thickness and then stained with H&E. Mammary gland tumors classification, and grade was classified as previously described (Cassali et al., 2020; Elston and Ellis, 1998). In case of multiple lesions, the one with worst prognosis by histological examination was considered.

### Serum biomarkers

Peripheral venous blood samples were obtained from all subjects during the preoperative stage and routine checkups for (control group). After the collection and clot retraction, the samples were centrifuged at 1,450g for 5 min. Serum tests were performed according to the manufacturer’s protocol. LDH serum activity was assessed on the date of collection, considering the kit recommendations regarding enzyme stability, and remaining serum was stored at -80 °C to perform the CRP and CA 15-3 assays after the histopathological diagnoses were conducted in less than 30 days.

All assays were performed in duplicate, and a positive and negative control (supplied with the kit) with a known concentration range were included. Labmax Plenno (Labtest*®*, Vista Alegre, Brazil) was used for obtaining the reaction readings of LDH and CRP.

For LDH dosage (U/L), a commercial kit was used (LDH Liquiform, Labtest*®*, Vista Alegre, Brazil) with a continuous ultraviolet kinetics method based on the reaction involving the conversion of pyruvate to lactate in the presence of NADH.

CRP was dosed (mg/L) using a commercial kit (PCR Turbiquest, Labtest*®*, Vista Alegre, Brazil) that operates on the principle of immunoturbidimetry using latex particles that are stabilized and sensitized with anti-CRP antibody.

The results of LDH and CRP were compared to the reference values established for the canine species (Carney et al., 2011; Kaneko et al., 2008).

CA 15-3 (IU/mL) levels were dosed using a commercial kit (BR-MA, Simens®, Munich, Germany), with an enzyme-linked fluorescent testing method (ELFA) using a two-step conjugation technique that conducts automated dosing with the IMMULITE 1000 Immunoassay System (Siemens®, Munich, Germany) with a measurement range of 2 to 400 U/mL. Up to the present date, there are no determined reference intervals for serum levels of CA 15-3 in healthy female dogs.

### Statistical analysis

The study considered each dog as an individual experimental unit for assessing CA 15-3, CRP, LDH, and clinicopathological variables.

Statistical analysis was performed using the R software, version 4.0.2 (R® foundation for statistical computing, Austria). Analysis of variance (ANOVA) was followed by Tukey’s tests for comparison of means between the groups. Possible correlations between clinical pathological parameters were evaluated using Pearson or Spearman test. The cut-off point, area under curve (AUC), sensitivity and specificity were calculated using the receiver operating characteristic (ROC) curve analysis in a logistic regression model to assess and compare the diagnostic performance of each biomarker. All statistical tests were considered significant when the test significance probability P<0.05.

## Results

### Characteristics of the dogs

Although breed of the dog was not used as a selection criterion, mixed breed dogs (07/15; 46.7%) and Poodles (04/15; 26.7%) were the most common in CMNs group. In control group, high frequency of Australian Shepherd (04/15) and Pembroke Welsh Corgi breed (03/15) was observed. Regarding histopathological features, mixed tumor carcinoma was the most frequently diagnosed (06/15, 40.0%), followed by tubular carcinoma (05/15, 33.33%), benign mixed tumor (03/15, 20.0%), and micropapillary carcinoma (01/15, 6.7%). The female dog with micropapillary carcinoma exhibited a clinical presentation of inflammatory carcinoma, and was euthanized. All the others survived until the end of the study. Distant metastases were found in three subjects with malignant CMNs (two with tubular, and one with micropapillary carcinoma). In the CMNs group, five malignant presentations were ulcerated (three non-metastatic and two metastatic). The mean size of all tumors was 6.1 ± 7.7 cm (Table 1).

**Table 1:** Description of the pathological clinical parameters observed in canine mammary tumor (CMT) group.

**Histopathology**	**Previously castration**	**Number of nodules**	**Size (cm)**	**Ulceration**	**Grade**	**Lymph node metastasis**	**Clinical stage**	**Breed**
Benign mixed tumor	No	2	0.8	No	-	No	I	Brazilian terrier
	No	2	3.5	No	-	No	II	Poodle
	No	2	6.5	No	-	No	III	Mixed breed
Micropapillary carcinoma	No	2	8.0	Yes	III	Yes	V	Mixed breed
Mixed tumor carcinoma	Yes	3	1.5	No	I	No	I	Poodle
	No	1	2.5	No	I	No	I	Mixed breed
	Yes	1	3.5	No	I	No	II	Poodle
	No	1	5.5	No	I	No	III	Labrador Retriever
	No	3	7.8	Yes	I	No	III	Mixed breed
	No	2	10.0	Yes	I	No	III	Mixed breed
Tubular carcinoma	No	1	1.0	No	I	No	I	Mixed breed
	No	3	1.5	No	II	No	I	Poodle
	No	5	3.8	Yes	I	No	I	Mixed breed
	Yes	1	3.8	No	II	Yes	V	Golden Retriever
	No	3	32.0	Yes	II	Yes	V	Golden Retriever

### Serum biomarkers evaluation

The average levels of CA 15-3, CRP, and LDH biomarkers in subjects with CMNs were 10.0 ± 4.3 IU/mL, 4.0 ± 2.0 mg/L, and 425.0 ± 244.4 U/L, respectively. In healthy subjects, the average levels of CA 15-3, CRP, and LDH were 1.0 ± 0.0 IU/mL, 1.0 ± 1.2 mg/L, and 299.0 ± 170.3 U/L, respectively. The dosages of CA 15-3 in all subjects in the control group were below the detection limit. For statistical purposes, these values were considered equal to 1 U/mL.

**Figure 1:**
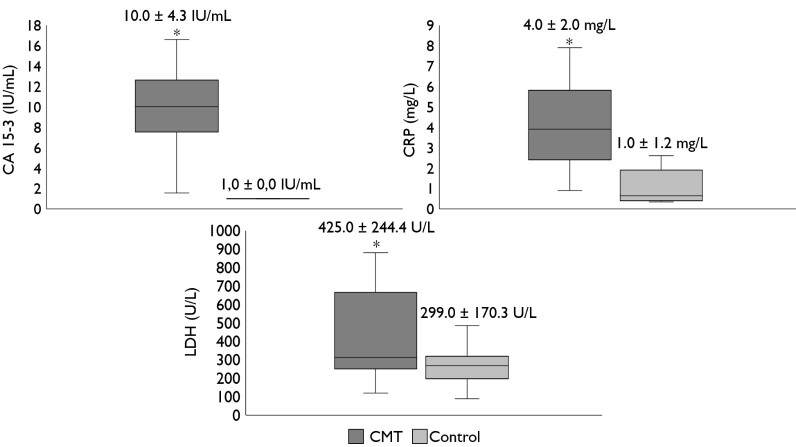
Serum CA 15-3, CRP and LDH concentrations in canine mammary neoplasms (CMNs) and in the control group. *Statistical difference (P < 0.05).

The mean levels of CA 15-3 (P<0.0001), CRP (P<0.0008), and LDH (P<0.02) were significantly different between the groups, showing that the subjects with CMNs exhibited significantly higher levels of CA 15-3, CRP, and LDH values, when compared with the control group (Figure 1).

### Diagnostic value of serum biomarkers

**Figure 2:**
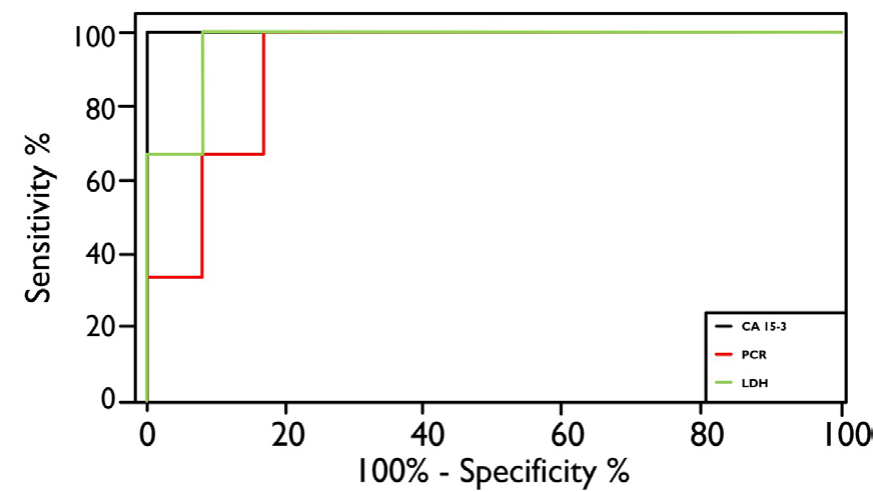
Receiver-operating characteristic curve comparing predictive sensitivity (%) and specificity (100-%) of CA 15-3, CRP and LDH serum biomarkers in canine mammary neoplasms (CMNs).

The best cut-off value was estimated to maximize the sum of sensitivity and specificity to diagnose an animal with mammary gland tumor. The CA 15-3 serum cut-off value established from the ROC curve was 1.5 IU/mL with 100.0% sensitivity, specificity, and AUC. CRP serum level cut-off determined using the ROC curve was 2.3 mg/L with 100.0% sensitivity, 91.7% specificity, and 97.2% AUC. LDH serum level cut-off determined using the ROC curve was 440 U/L with 100.0% sensitivity, 46.7% specificity, and 73.9% AUC (Figure 2).

Neither CRP nor LDH were affected by the number of nodules. However, the levels of CA 15-3 were significantly affected by the number of nodules in the mammary gland (r = 0.68). CA 15-3, CRP, and LDH levels were positively correlated with ulceration, tumor size, histopathological grade, metastatic lymph node, and clinical staging. Additionally, CRP concentration were positively correlated with the total plasma protein dosage (r = 0.52), and CA 15-3 concentration was positively correlated with CRP (r = 0.72) as well as LDH values (r = 0.54) (Table 2).

**Table 2:** Correlations observed between prognostic parameters and the serum biomarkers CA 15-3, CRP and LDH evaluated in canine mammary tumors (CMT).

**Biomarker**	**Number of nodules**	**Ulceration**	**Tumor size**	**Histopathological grade**	**Lymph node metastasis**	**Clinical staging**	**Plasma protein (g/dL)**	**CRP (mg/L)**		**LDH (U/L)**
CA 15-3*	r = 0.68	r = 0.59	r = 0.64	r = 0.90	r = 0.60	r = 0.87	-	r = 0.72	r = 0.54	
CRP*	-	r = 0.42	r = 0.58	r = 0.59	r = 0.61	r = 0.85	r = 0.52	r = 1.0		-
LDH*	-	r = 0.62	r = 0.54	r = 0.59	r = 0.66	r = 0.52	-	-		r = 1.0

† r = Value by Pearson or Spearman test. * Statistical difference (P < 0.05).

## Discussion

Biomarkers of human breast cancer can be detected in CMNs (Kaszak et al., 2018). Western blotting analysis was performed to confirm the specificity and possible cross-reactivity of human CA 15-3, suggesting a good interaction between human and canine MUC-1, thereby enabling the application of human reagent kits in dogs (Campos et al., 2012). Also, CRP and LDH have already been determined in canine cases using human kits (Campos et al., 2012; Kjelgaard-Hansen et al., 2003).

The results indicate a statistical difference in serum CA 15-3 concentration between groups with and without CMNs, as many studies shown (Baba et al., 2019; Campos et al., 2012, 2015; Manuali et al., 2012; Marchesi et al., 2010), illustrating a potential clinical use of CA 15-3 in cases of CMNs.

Our results also suggest that the mean concentration of CRP was significantly higher in subjects with CMNs (4.0 ± 1.9 mg/L) when compared to those in the control group (1.0 ± 1.2 mg/L), a result that is similar to previously study, where higher values of CRP were found in mammary gland carcinomas (median 4.7 mg/L, range 0.63 to 128.96) compared to control group (median 2.1 mg/L, range 0.25 to 6.57) (Planellas et al., 2009).

LDH has been described as a good biomarker in canine tumors, especially for predicting the recurrence in lymphomas (Marconato et al., 2009, 2010). The study conducted by Campos et al. (2012) is one of the few wherein LDH concentration in CMNs. Similar results were found in the present work.

Valencakova-Agyagosova et al. (2014) proposed a cut-off value of 5.0 to 7.0 IU/mL, with 100% sensitivity and 95.0% specificity, and suggested the application of CA 15-3 as the first choice biomarker given the considerable sensitivity and specificity found for CMNs. Campos et al. (2012) proposed a cut-off of 0.50 IU/mL; however, the sensitivity and specificity values were not reported in this work. These results differ from those obtained in this study (cut-off 1.5 IU/mL), which might be due to the different methodologies and reagents used. No reference ranges indicated for CA 15-3 were suggested to this date, and thus should be determined in future studies.

Neither of the works who evaluate CRP (Planellas et al., 2009; Szczubiał et al., 2018) and LDH (Campos et al., 2012) in canine females with CMNs indicate cut-off points. Since CRP and LDH exhibit non-specific increase in various situations (Carney et al., 2011; Kaneko et al., 2008), they could not be applied individually as CMNs biomarkers. Although this lack of specificity, they may serve as the cofactors to increase the diagnostic accuracy to detect CMNs, given their concentration’s correlation with CA 15-3, as determined in the present study.

In addition, CA 15-3 exhibited higher AUC, sensitivity, and specificity than CRP and LDH, indicating that CA 15-3 is a more powerful biomarker for CMNs (Figure 2). It is probably because this biomarker is an associated antigen to cancer, present in higher concentrations in neoplasms. CA 15-3 detects the proteolytically cleaved soluble form of Mucin 1 (MUC1) which in cancer cells are hypoglycosylated and with polarity loss, thereby acting as anti-adhesive molecules and enabling the detachment of malignant cells. This increases the release of MUC1 into the circulation where it can be measured using the immunoassays (David et al., 2016; Hollingsworth and Swanson, 2004).

Patients with largest tumors, lymph node metastasis (Campos et al., 2012; Campos et al., 2015) and higher grades based on histopathological analysis (Manuali et al., 2012) exhibit significantly higher concentration of CA 15-3. CMNs serum samples associated with microscopical inflammation do not seem to influence the levels of CA 15-3 (Marchesi et al., 2010). In our study, female dogs with macroscopic ulceration of the nodules exhibited higher values of CA 15-3, probably because tumors with aggressive behavior are usually ulcerated (Sorenmo et al., 2020).

When it comes to the clinical staging, a link between CA 15-3 levels and the presence of distant metastasis was observed, in which female dogs with the highest clinical staging were those that presented the highest CA 15-3 concentrations.

Regarding the CRP levels, female dogs which had larger nodules (Szczubiał et al., 2018), ulcerated skin (Planellas et al., 2009; Szczubiał et al., 2018), distant metastasis (Szczubiał et al., 2018), and high clinical stages (Tecles et al., 2009) exhibited higher concentrations of CRP.

Campos et al. (2012) demonstrated a positive correlation only between LDH concentration and clinical staging. To the best of our knowledge, our study is the first work to determine a positive correlation between ulceration, tumor size, histopathological grade, metastatic lymph node, and LDH concentration to date, also the first to find a positive correlation between the histopathological grade and CRP serum concentration.

As previously stated, the number of nodules, tumor size, ulceration, histopathological grade, lymph node metastasis and clinical staging are all associated with worst prognosis in female dogs with CMNs (Araújo et al., 2016; Cassali et al., 2020; Ferreira et al., 2009; Nunes et al., 2018a, b). As in the results, each of the evaluated serum biomarkers are related with more than one of the prognostic factors reported, we believe that the use of CA 15-3, CRP and LDH combined in follow-ups can be done in order to provide a low-cost prognostic information follow-up in cases of CMNs.

The association of CRP and LDH with other biomarkers have a high sensitivity in the detection of metastatic human breast cancer. Unfortunately, in cases of CMNs, the biomarker panels that relate to the previously described prognostic parameters and may be able to determine the patient’s outcome are poorly described. So far, serum biomarkers are not indicated for primary diagnosis of CMNs (Kaszak et al., 2018). Further studies are needed to evaluate of these same serum biomarkers nder the same conditionsu.

Serum concentration of CA 15-3 decreased significant post-surgery (Roberto et al., 2018), suggesting that an increase in their value after mastectomy can be used as evaluation of tumor recurrence. Furthermore, a lower mean survival in CMNs with higher serum concentrations of CA 15-3 has been reported (Baba et al., 2019). The association of the previously studies to ours indicates that the use of a biomarker panel that includes CA 15-3, CRP and LDH in cases of CMNs would improve patient follow-up monitoring.

## Conclusion

In conclusion, female dogs with CMNs were found to exhibit highest serum levels of CA 15-3, CRP, and LDH. Therefore, this panel could be applied to improve the efficacy of the diagnosis of CMNs as well as the prognostic evaluation in female dogs. However, further studies are still required to be conducted with large number of animals, serial dosage before and after mastectomy, and with a larger follow-up period to confirm the diagnostic value in such conditions. 
